# Manipulation of Magnetic Skyrmion Density in Continuous Ir/Co/Pt Multilayers

**DOI:** 10.3390/mi13111911

**Published:** 2022-11-04

**Authors:** M. Cubukcu, S. Pöllath, S. Tacchi, A. Stacey, E. Darwin, C. W. F. Freeman, C. Barton, B. J. Hickey, C. H. Marrows, G. Carlotti, C. H. Back, O. Kazakova

**Affiliations:** 1 National Physical Laboratory, Teddington TW11 0LW, UK; 2 London Centre for Nanotechnology, University College London, 17-19 Gordon Street, London WC1H 0AH, UK; 3 Institut für Experimentelle Physik, Universität Regensburg, D-93040 Regensburg, Germany; 4 Istituto Officina dei Materiali del CNR (CNR-IOM), Sede Secondaria di Perugia, c/o Dipartimento di Fisica e Geologia, Università di Perugia, I-06123 Perugia, Italy; 5 School of Physics and Astronomy, University of Leeds, Leeds LS2 9JT, UK; 6 Dipartimento di Fisica e Geologia, Università di Perugia, Via Pascoli, I-06123 Perugia, Italy; 7 Physik-Department, Technical University Munich, 85748 Garching, Germany

**Keywords:** skyrmions, Dzyaloshinskii–Moriya interaction, spintronics

## Abstract

We show that magnetic skyrmions can be stabilised at room temperature in continuous [Ir/Co/Pt]_5_ multilayers on SiO_2_/Si substrates without the prior application of electric current or magnetic field. While decreasing the Co thickness, a transition of the magnetic domain patterns from worm-like state to separated stripes is observed. The skyrmions are clearly imaged in both states using magnetic force microscopy. The density of skyrmions can be significantly enhanced after applying the “in-plane field procedure”. Our results provide means to manipulate magnetic skyrmion density, further allowing for the optimised engineering of skyrmion-based devices.

## 1. Introduction

Low-dimensional topological spin textures in magnetic materials are technologically attractive since it is expected that they can be used in next-generation storage devices as information carriers. One example is the magnetic skyrmion—a nanometre-sized, topologically protected, swirling spin texture. Their topological properties and efficient current-driving dynamics, together with their nanoscale size and stable particle-like features, make magnetic skyrmions promising candidates for carrying magnetic information in future high-density and low-power consumption spintronic devices [1,2,3,4].

Recently, heavy metal (HM)/ferromagnet (FM) multilayers deposited by magnetron sputtering and hosting skyrmions have attracted attention. Indeed, the strong spin–orbit coupling (SOC) of the HM layer can lead to an antisymmetric exchange known as the interfacial Dzyaloshinskii–Moriya interaction (iDMI) [5,6], which plays a key role in the stabilisation of magnetic skyrmions [7,8,9,10]. Regarding this, different combinations of HM/FM multilayers, such as Ta/Co/Pt, Ir/Co/Pt, and W/Co/Pt, have been intensively investigated. The generation of room-temperature skyrmions in these multilayers has been observed, as well as their motion under spin torques [11,12]. When iDMI is utilised for the formation of skyrmions, it is possible to control the nucleation processes and skyrmions’ properties using a variety of approaches [13,14]. In this way, relevant magnetic parameters, such as the perpendicular magnetic anisotropy (PMA) or the iDMI strength, can be strongly modified to affect both their nucleation and properties (e.g., density, size, and dynamics).

Although skyrmions have been observed at room temperature in HM/FM multilayers, in most cases, their nucleation and stabilisation require an injection current and/or external magnetic field [15,16,17]. Additionally, lithographically defined structures were used to confine single or multiple skyrmions depending on the geometry [18,19]. The usage of skyrmions in the next-generation spintronic devices will depend on the achievement of skyrmions without the support of external stimuli. Hence, it is important to nucleate skyrmions without the need for any external force, even without nanostructured confinement. In this aspect, the search for an efficient method to establish skyrmions yields interesting findings, such as the observation of zero-field skyrmions at room temperature through an exchange bias field created at the interface of the antiferromagnetic/ferromagnetic-based structure [20,21] and their direct writing using X-rays [22] and electron beams [23]. Moreover, the investigations into how to enhance the skyrmion density are of great significance for achieving ultrahigh density spintronics devices. For example, the skyrmion density in [Ta/Co/Pt]_n_ has been enhanced by changing the Co thickness [24], and the crossover from a few isolated skyrmions to a dense skyrmion lattice has been realised by controlling the Co and Fe composition in [Ir/Fe/Co/Pt]_n_ [25]. The influence of structure repetition (n) on skyrmion density in [Pt/Co/Ta/MgO]_n_ has also been investigated [26]. Additionally, it has been shown that the in-plane magnetic field contributes to the creation of skyrmions in [Ta/Co/Pt]_n_ and that a high concentration of skyrmions can be achieved by increasing the in-plane field [27]. In fact, the skyrmion density increased with increasing the critical material parameter *κ*$=\pi D/4\sqrt{AK_{eff}}$, where *A* is the exchange stiffness, $K_{eff}$ is the effective PMA, and *D* is the iDMI constant [25]. The application of the in-plane field will diminish the role of the PMA, while keeping iDMI and *A* unchanged, which leads to the increase of *κ* [27].

In this work, we show that magnetic skyrmions can be stabilised at room temperature in continuous [Ir/Co/Pt]_5_ multilayers on SiO_2_/Si substrates; external magnetic fields, current injections, and geometric confinement are not required to generate skyrmions. The magnetic, structural, and interfacial parameters of the multilayer are analysed using vibrating sample magnetometry, X-ray reflectivity, and Brillouin light scattering. The imaging of skyrmions was performed by magnetic force microscopy. By thinning the Co layer (*t_Co_*) a transition of the magnetic domain patterns from a worm-like state to separated stripes is observed. The skyrmions are clearly observed in both states. We also report that the density of skyrmions can be significantly enhanced after undergoing an “in-plane field procedure”, in which a high density of skyrmions can be detected after applying an in-plane magnetic field of around 2 T and subsequently ramping it down to zero. Magnetisation curves showed the dependence of the perpendicular magnetic anisotropy (PMA) with the Co thickness, providing a way to interpret the magnetic textures observed in the magnetic force microscopy images. These results could provide a criterion for designing skyrmion magnetic thin films, which has the potential to advance the development of skyrmion-based magnetic devices.

## 2. Sample Fabrication and Characterisation

The multilayers [Ir(1.2 nm)/Co(*t_Co_*)/Pt(1.3 nm)]_5_ and (*t_Co_* = 0.4–0.8 nm) were grown using DC magnetron sputtering in a high-vacuum system. The samples grown on SiO_2_/Si substrates were used to determine the magnetic, structural, and interfacial properties using vibrating sample magnetometry (VSM), Brillouin light scattering (BLS), and X-ray reflectivity (XRR), as well as to image their magnetic textures using magnetic force microscopy (MFM). Additionally, the identical counterpart multilayers grown on a Si_3_N_4_ membrane (deposited in the same run) were used for Lorentz transmission electron microscopy (LTEM) measurements (see Supplementary Materials). The base pressure in the chamber before growth was of the order of 1 × 10^−8^ mbar, and a flow of 60 sccm/5.02 mTorr of argon gas was used throughout the sputtering process. The different layers in the multilayer structure were grown in turn by moving the substrate over the top of the sputter guns for set periods of time, while applying a constant source current to the target materials. The target composition, gun position, source current, and subsequent power of the magnetron gun for each material are shown in Table S1 (see Supplementary Materials), as well as the typical growth rates for each material. The separation between the sputter target and the sample substrate was 7 cm during growth.

**Figure 1:**
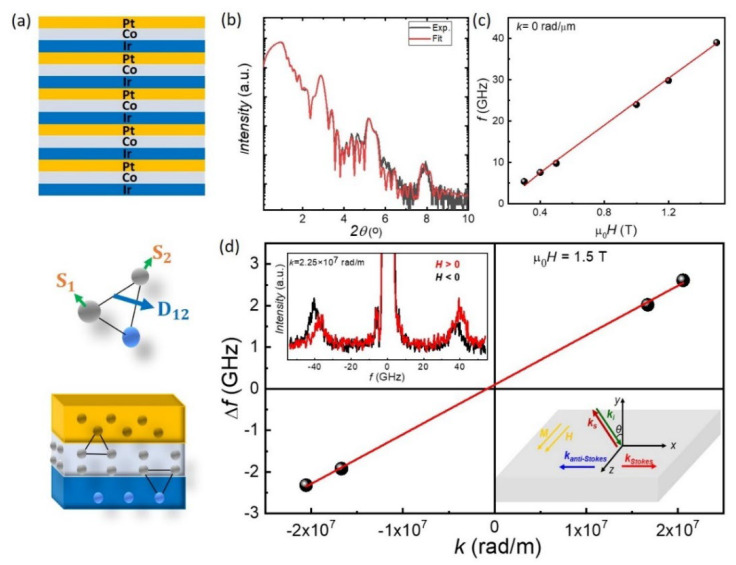
Structural and interfacial characterisation of [Ir/Co/Pt]_5_ multilayers for *t_Co_* = 0.8 nm at room temperature. (**a**) Schematic diagrams of the multilayer film structure and the corresponding interfacial DMI produced between S_1_ and S_2_ spins of two adjacent Co atoms located close to the Ir or Pt atoms with a strong SOC. (**b**) XRR measurement result: intensity as a function of 2θ incident angle. (**c**,**d**) BLS measurements. (**c**) Dependence of the spin wave (SW) frequency (*f*) as a function of the intensity of the in-plane applied field, $\mu_{0}H$, as measured at normal incidence for *k* = 0 rad/µm. (**d**) SW frequency asymmetry, Δf, measured at *k* = 1.67 × 10^7^ rad/m and *k* = 2.25 × 10^7^ rad/m when reversing the direction of the applied magnetic field, which is equivalent to the reversal of the propagation direction of the DE mode. Top inset: BLS spectra measured at *k* = 2.25 × 10^7^ rad/m. Bottom inset: Schematic of BLS experiment. The sample is saturated in-plane by an external field, $\mu_{0}H$ = 1.5 T, applied along the z-axis. Stokes and anti-Stokes events in the scattering process correspond to SW propagating with +k and −k, respectively.

The multilayer structure is schematically illustrated in Figure 1a (top). The sample structure was characterised using XRR (Figure 1b) and the resulting fringe pattern was simulated using GenX [28] confirming the thicknesses within the multilayer. The fitting parameters are shown in Section S1 (see Supplementary Materials).

In the [Ir/Co/Pt]_5_ multilayer film, there is iDMI between spins S_1_ and S_2_ of two adjacent Co atoms located close to heavy metals atoms (Ir or Pt) with a strong SOC. The Hamiltonian can be expressed as H_DMI_ = D_12_·(S_1_ × S_2_) [29], where D_12_ is the DMI vector as shown in Figure 1a (bottom). To determine the strength of the iDMI, we used BLS. BLS measurements from thermally excited spin waves (SWs) were performed in the backscattering geometry focusing about 150 mW of a monochromatic laser beam (wavelength λ = 532 nm) on the sample surface through a camera objective with numerical aperture NA = 0.24. The frequency of the scattered light was analysed by a Sandercock-type (3 + 3)-tandem Fabry-Perot interferometer (The table stable ltd, Mettmenstetten, Switzerland). Due to the conservation of momentum in the light-scattering process, the magnitude of the spin wave vector *k* is related to the incidence angle of light *θ*, by the relation *k* = 4π sin *θ*/λ. First, the dependence of the SW frequency as a function of the intensity of the in-plane applied field $\mu_{0}H$ was measured at normal incidence, i.e., for *k* = 0 rad/m (Figure 1c, dots). To quantitatively estimate the out-of-plane anisotropy constant $K_{u}$ and the gyromagnetic ratio γ, a best fit procedure of the experimental data (Figure 1c, red line) was performed using the Kittel equation:

(1) $${\left(\frac{\omega }{\gamma }\right)}^{2}=\left[H\cdot \left(H-\frac{2}{M_{S}}K_{u}+4\pi M_{S}\right)\right]$$

where *M_s_* is the saturation magnetisation of the ferromagnet [30]. From this analysis the values $K_{u}=$1.89 × 10^6^ J/m^3^ and γ = 176 GHz/T were obtained for the *t_Co_* = 0.8 nm sample. The strength of the iDMI was quantitatively extracted by measuring the iDMI induced frequency asymmetry, Δf, for Damon–Eshbach (DE) modes propagating in opposite directions. BLS measurements were performed in the DE geometry, applying an in-plane magnetic field $\mu_{0}H$=1.5 T sufficiently large to saturate the magnetisation in the film plane, and sweeping the in-plane transferred wave vector along the perpendicular direction. The top inset of Figure 1d shows the BLS spectra measured at *k* = 2.25 × 10^7^ rad/m. The Stokes and anti-Stokes peaks are characterised by a sizeable frequency asymmetry, which reverses upon reversing the magnetic field direction. Figure 1d shows the SW frequency asymmetry, Δf, measured at *k* = 1.67 × 10^7^ rad/m and *k* = 2.25 × 10^7^ rad/m upon reversing the direction of the applied magnetic field, which is equivalent to the reversal of the propagation direction of the DE mode. The effective iDMI constant, *D*, was determined by means of a linear fit (continuous red line) to the experimental data using the relation $\Delta f=\frac{2\gamma D}{\pi M_{s}}k$, and fixing the gyromagnetic ratio and the saturation magnetisation to the values obtained from the analysis of the BLS measurements as a function of $\mu_{0}H$ and from VSM measurements, respectively. A value for *D* was obtained, $D=\left(1.8\pm 0.2\right)\mathrm{mJ}/m^{2}$, indicating that the right-handed chirality is favoured by the iDMI. This is in agreement with previous investigations that expect a right-handed chirality for a Co/Pt stack where the Pt is the overlayer [31].

## 3. Results and Discussion

**Figure 2:**
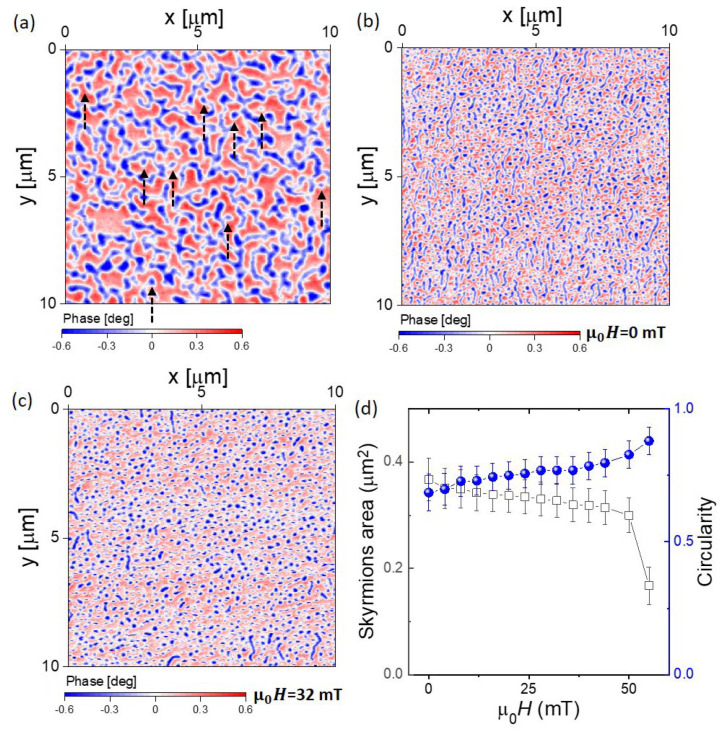
MFM measurements on [Ir/Co/Pt]_5_ multilayers for *t_Co_* = 0.8 nm at room temperature. (**a**) The MFM image was acquired in the as-grown state. Red and blue contrast represents out-of-plane magnetisation of opposite directions. Some skyrmions are indicated by dashed black arrows. (**b**) Magnetic state following the “in-plane field procedure”. (**c**) Example of the evolution of skyrmions vs. the perpendicular applied magnetic field at $\mu_{0}H=32\;mT$. (**d**) Plot showing the area of the skyrmions (open square) and circularity (blue spheres) vs. $\mu_{0}H$.

The configuration of the vertical magnetic texture was investigated with MFM. The MFM imaging of the multilayers was performed at room temperature with an NT-MDT Ntegra Aura (Moscow, Russia) scanning probe microscope (SPM) [31,32,33,34]. The system is fitted with an electromagnet, which allows the application of an out-of-plane magnetic field up to 115 mT during scans. Low moment tips (NT-MDT MFM-LM) were chosen to minimise the probe–sample interaction. All MFM images were obtained using the lift mode at a pre-set lift height of 100 nm. To image the magnetic domain patterns without any prior applied magnetic field, the samples were imaged in the as-grown state when *t_Co_* = 0.8 nm (Figure 2a). The MFM images show that the magnetisation is broken up into small domains of a worm-like configuration. Some skyrmions were also clearly observed among the worm-like textures, as indicated by the dashed black arrows in Figure 2a. As the measurements were performed before cycling the magnetic field, these images reveal that no prior stabilizing magnetic field or injection current are required to generate skyrmions. Therefore, skyrmions at zero field can be spontaneously stable, even for samples in the as-grown state. Then, in order to explore the different processes that can stabilise skyrmions or/and manipulate the density of skyrmions, the sample was imaged after applying an in-plane magnetic field of around 2 T and subsequently turning off the in-plane magnetic field (Figure 2b). We refer to this sequence as the “in-plane field procedure”. In fact, in previous investigations, it has been reported that the applied in-plane component of the magnetic field will affect the concentration of skyrmions [27]. In Figure 2b, we show the MFM images at zero field after application of the “in-plane field procedure”. This procedure is highly favourable for skyrmion formation and increases their density, creating a maximum skyrmion area value of ≈0.37 μm^2^ (before it was ≈0.03 μm^2^). The skyrmion area is defined from the area of the data that are extracted using a 50% threshold and is implemented using image processing. After the “in-plane field procedure”, MFM images were obtained under different applied out-of-plane magnetic fields, $\mu_{0}H$. An example of the images at $\mu_{0}H=32\;mT$ is shown in Figure 2c. In Figure 2d, we show the dependence of the skyrmions’ area on $\mu_{0}H$. We show that the skyrmions’ area slightly decreases (ranging from ≈0.37 μm^2^ to ≈0.3 μm^2^) at low $\mu_{0}H$, and then a sharp decrease occurs at high $\mu_{0}H$ (down to ≈0.17 μm^2^) before the magnetisation reaches the saturation point. The circularity is almost constant at low $\mu_{0}H$ and increases slightly when $\mu_{0}H$ is increased (ranging from ≈0.7 to ≈0.9). The circularity is defined by fitting an ellipse to these extracted data; then, we take the ratio of the semi minor and semi major axis of the ellipse (i.e., 1 to 1 is a circle and 0.5 to 1 is an ellipse).

**Figure 3:**
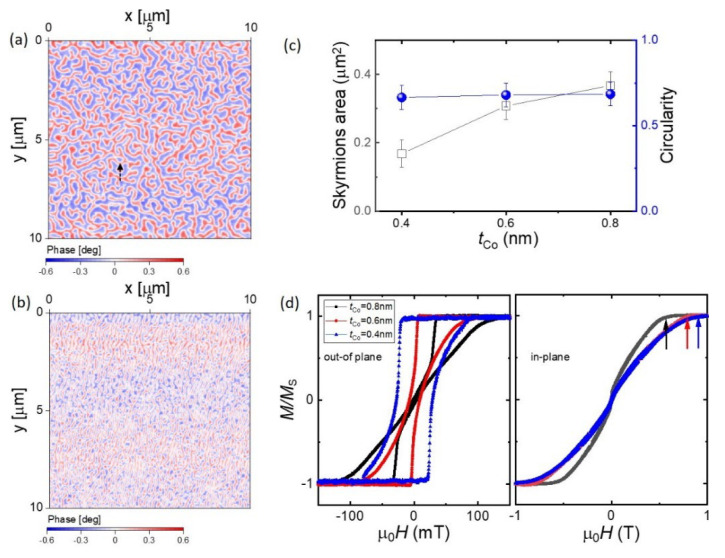
MFM and magnetisation measurements vs. Co thickness (*t_Co_*) at room temperature. The MFM images were acquired in the as-grown state for *t_Co_* = 0.6 nm (**a**) and *t_Co_* = 0.4 nm (**b**). (**c**) Plot showing the area of the skyrmions and the circularity vs. *t_Co_* at zero field after the “in-plane field procedure”. (**d**) Normalised hysteresis curves, *M*/*M_S_* vs. the external magnetic field $\mu_{0}H,$ in both the out-of-plane (left) and in-plane (right) directions for *t_Co_* = 0.8 nm, *t_Co_* = 0.6 nm, and *t_Co_* = 0.4 nm. Arrows show the anisotropy field, which is obtained from the in-plane magnetisation curve at saturation.

We further studied the effect of the Co thickness on the magnetic properties of the [Ir/Co(*t_Co_*)/Pt]_5_ multilayers (Figure 3). For *t_Co_* = 0.6 nm, the magnetic domains exhibit a clear worm-like configuration, though some individual skyrmions can be seen in the as-grown state (Figure 3a). By further reducing the Co layer thickness (*t_Co_* = 0.4 nm), we observed a transition from the worm-like pattern to separate stripes in the magnetic domain (Figure 3b). By reducing the Co thickness to 0.4 nm, we observed a smaller size of skyrmions in the as-grown state (Figure 3b). In addition, in Figure 3c, we show the dependence of the skyrmions’ area versus *t_Co_* at zero field after the “in-plane field procedure”. The skyrmions’ area decreases with decreasing *t_Co_* (ranging from ≈0.37 μm^2^ to ≈0.18 μm^2^) and the circularity remains almost constant (≈0.7). To understand the effect of thickness, we refer to the magnetisation measurements. The out-of-plane and in-plane magnetisation curves (normalised to the saturation magnetisation *M_s_*) are summarised in Figure 3d. For the thicker Co samples (0.8 nm and 0.6 nm), the out-of-plane hysteresis shows a tail feature, whilst the thinner sample (0.4 nm) presents a more square-shaped loop. The anisotropy field (see arrows), which is obtained from the in-plane magnetisation curve at saturation, is higher for the samples with thinner Co layers, indicating an increased PMA [35].

## 4. Conclusions

In summary, we investigated the formation of magnetic domains in [Ir/Co/Pt]_5_ multilayers. The magnetic skyrmions can be stabilised at room temperature without the prior application of either an electric current or magnetic field. By reducing the Co thicknesses, we observed a transition from a worm-like magnetic domain pattern to separate stripes. The skyrmions are also clearly observed in both states. Significantly, a high density of skyrmions is imaged after undergoing the “in-plane field procedure”. Our results could provide a criterion for designing a skyrmion magnetic thin film, which may advance the development of skyrmion-based magnetic devices.

## Data Availability

Data associated with this work are available from the University of Leeds repository at https://doi.org/10.5518/1262.

## Supplementary Materials

The following supporting information can be downloaded at: https://www.mdpi.com/article/10.3390/mi13111911/s1, Section S1: Growth Conditions; Table S1: The target materials and sputtering parameters used to grow the magnetic multilayers; Section S2: LTEM Zero-Field Cooling Measurements; Figure S1: LTEM study of [Ir/Co/Pt]_5_ multilayers for *t_Co_* = 0.8 nm. Section S3: LTEM Field-Polarized Cooling Measurements; Figure S2: The phase diagram resulting from a field-polarized cooling procedure on multilayers for *t_Co_* = 0.8 nm. References [36,37] are cited in the supplementary materials.
