# Supplementary material for: Manipulation of Magnetic Skyrmion Density in Continuous Ir/Co/Pt Multilayers
Source: Micromachines (Basel). 2022 Nov 4;13(11):1911. doi: 10.3390/mi13111911 (PMC9693305; doi:10.3390/mi13111911)
Supplement: Supplementary file 1 [file micromachines-13-01911-s001.zip › micromachines-1974460-Supplementary.pdf]

micromachines

Supplementary material

Manipulation of magnetic skyrmion density in continuous Ir/Co/Pt multilayers

M. Cubukcu [1,2*], S. Pöllath [3], S. Tacchi [4], A. Stacey [5], E. Darwin [5], C. W. F. Freeman [1,2], C. Barton [1], B. J. Hickey [5], C. H. Marrows [5], G. Carlotti [6], C. H. Back [7] and O. Kazakova [1]

1 National Physical Laboratory, Teddington, TW11 0LW, UK
2 London Centre for Nanotechnology, University College London, 17-19 Gordon Street, London WC1H 0AH, UK
3 Institut für Experimentelle Physik, Universität Regensburg, D-93040 Regensburg, Germany
4 Istituto Officina dei Materiali del CNR (CNR-IOM), Sede Secondaria di Perugia, c/o Dipartimento di Fisica e Geologia, Università di Perugia, I-06123 Perugia, Italy
5 School of Physics and Astronomy, University of Leeds, Leeds LS2 9JT, UK
6 Dipartimento di Fisica e Geologia, Università di Perugia, Via Pascoli, I-06123, Perugia, Italy
7 Technical University Munich, 85748 Garching, Germany
* Correspondence: murat.cubukcu@npl.co.uk

S1. Growth Conditions

The target materials and sputtering parameters used to grow the magnetic multilayers are shown in table S1. The sample structure was characterized using X-ray reflectometry (XRR) (Figure 1b in the main manuscript) and the resulting fringe pattern was simulated using GenX[1] confirming the multilayer structure is as follows:

Ta(18.75)/Ta_2O_5(6±5)/Pt(100±2)/Co(8±2)|Pt(13±2)/[Ir(11.77±0.6)/Co(8.8±0.7)/Pt(13.2±0.9)]$_{x5}$/Pt(32.9±0.3) where all thicknesses are provided in angstroms, Å, and the error on the Ta thickness found from the GenX fit is negligible[2]. The XRR fringes are shown in Figure 1(b) in the main manuscript, with the GenX fit included in Figure 1(b).

Table S1. The target materials and sputtering parameters used to grow the magnetic multilayers.

Target Material	Magnetron Gun Position	Source Current (mA)	Power (W)	Typical Growth Rate (Å/s)
Tantalum (Ta)	8	50	14	1.15
Platinum (Pt)	4	25	9	1.64
Cobalt (Co)	2	50	15	0.70
Iridium (Ir)	6	25	9	0.91

[1]GenX - https://aglavic.github.io/genx/
[2] ± 2 x10^{-6} Å.

S2. LTEM Zero-Field Cooling Measurements

As a final step in our investigation, using Lorentz transmission electron microscopy (LTEM, Tecnai-F30) we examined an $[Ir/Co/Pt]_5$ sample with Co layer thickness t_{Co}=0.8 nm deposited onto an electron beam transparent Si_3N_4 membrane under the same experimental condition (in the same run) as the previous samples in the main manuscript. Since we investigate Néel type skyrmions, the sample was tilted by 20° in all images [S1]. The temperature was set in a liquid nitrogen sample holder and controlled by using an installed heater. The multilayer was investigated in a temperature regime of approximately -175 to 75 °C. At all temperatures, the applied magnetic field $\mu_0 H$ was ramped from zero to 180 mT (after zero-field cooling). Figure S2(a) shows the phase diagram resulting from a zero-field cooling procedure. Here, a designated temperature was set and reached in zero magnetic field. Subsequently, the magnetic field was ramped

towards high fields and images were recorded, as indicated by the black dots in Figure S2(a). After the measurements, the sample was warmed up again, and a new target temperature gets set. In our LTEM images, we observe worm-like domains in most regions of the phase diagram below the critical field, where the sample becomes entirely field polarized. We denote this region 'the helical phase' (Hel) as it resembles the ordered helical phase in B20 bulk materials [S2]. This region is coloured green in Figure S2(a). Upon increasing the magnetic field, this domain state becomes scarce and is mostly replaced by the field polarized state (light green). Only in a very narrow region of magnetic field and temperature space (light blue), some (very few) skyrmions are observed in this sample, see the red box in Figure S2(b). Sample images from the different regions are shown in Figure S2(b-d): (b) mixed field polarized-skyrmion state with some residual worm domains, (c) worm domains and field polarized state, (d) field polarized state, (e) worm domain state in a close-to-zero magnetic field. The results obtained by LTEM are distinctly different from the MFM results and can be traced back to the use of a different substrate for the LTEM experiments. It is known that metallic films deposited onto thin, low stress Si_3N_4 substrates lead to a warping of the substrate that can significantly alter the magnetic state [S3] through stress induced anisotropy changes.

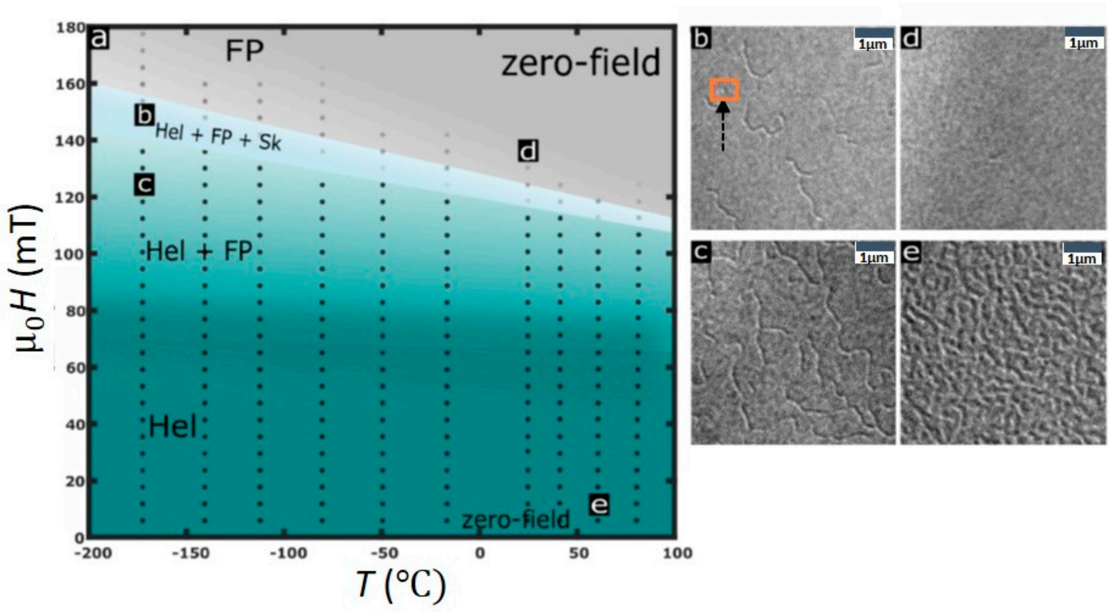

Figure S1. LTEM study of [Ir/Co/Pt]$_5$ multilayers for t_{Co}=0.8 nm. (a) The phase diagram resulting from a zero-field cooling procedure. (b-e) Sample images from the different regions as indicated in Figure 4(a): mixed field polarized-skyrmion state (FP+Sk) with some residual worm-domains (helical phase (Hel)) (b), worm-domains and field polarized state (FP) (c), FP state (d), worm-domain (Hel) state in close to zero magnetic field (e). A skyrmion is indicated by dashed black arrow.

S3. LTEM Field-Polarized Cooling Measurements

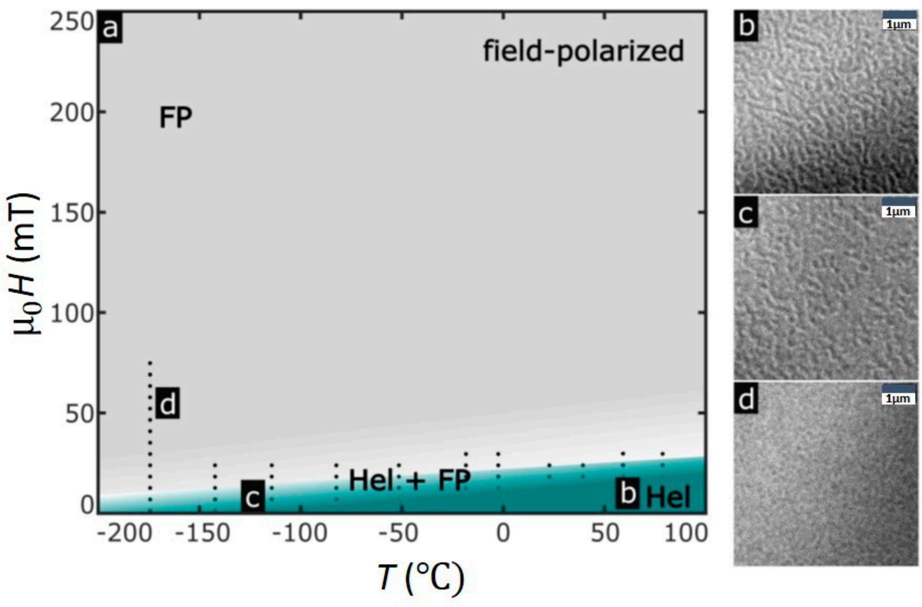

Figure S2. (a) The phase diagram resulting from a field-polarized (FP) cooling procedure on multilayers for t_{Co}=0.8 nm. Here, we field cool in a magnetic field of 250 mT and then reduce the field to zero. (b-d) The image series is taken from the different regions as indicated in Figure S3(a). It shows the hysteretic behaviour of the sample. In essence, we observe the field polarized (FP) state in most regions (d) in the magnetic field / temperature phase space and only close to zero magnetic field worm-like domains (b-c) are observed.

References

1. Benitez, M.J.; Hrabec, A.; Mihai, A.P.; Moore, T.A.; Burnell, G.; McGrouther, D.; Marrows, C.H. Magnetic microscopy and topological stability of homochiral Néel domain walls in a Pt/Co/AlO_x trilayer. *Nat. Commun.* **2015**, *6*, 8957.
2. Fert, A.; Cros, V.; Sampaio, J. Skyrmions on the track. *Nat. Nanotech.* **2013**, *8*, 152–156.
3. Dietrich, C.; Hertel, R.; Huber, M.; Weiss, D.; Schäfer, R.; Zweck, J. Influence of perpendicular magnetic fields on the domain structure of permalloy microstructures grown on thin membranes. *Phys. Rev. B* **2008**, *77*, 174427.
